# Lifestyle Medicine and the Role of the Pediatrician

**DOI:** 10.1007/s13312-025-00066-w

**Published:** 2025-05-12

**Authors:** Mahima Gulati, Suneela Garg

**Affiliations:** 1 Department of Medicine, Division of Endocrinology, Diabetes, and Metabolism, University of Connecticut Health School of Medicine, Farmington, CT USA; 2 Community Medicine, MAMC, Delhi Chair, Programme Advisory Committee, NIHFW, New Delhi, India

**Keywords:** Habit, Non-communicable diseases, Nutrition, Physical activity, Well-being

## Abstract

Non-communicable diseases (NCDs) are now the leading causes of human deaths worldwide. The current paradigm of ills, pills, drills and bills does not lend well to optimal management of NCDs. Indeed, what is required is an updated skills set, with the practitioner serving as a coach to the patient. The root cause of NCDs lies in dysregulated lifestyle behaviors and habits. Lifestyle medicine is the fastest growing medical specialty in the house of medicine. It aims to systematically implement the evidence-based science of healthy lifestyle habits. The six pillars of Lifestyle medicine are healthy, wholesome nutrition, regular physical activity, avoiding use of risky substances like tobacco, restorative sleep, stress management and resiliency, and fostering positive social relations (“forks, feet, fingers, sleep, stress, love”). This perspective article describes the competencies pediatricians can harness to apply Lifestyle medicine in their daily clinical practice.

## Introduction

India is the world’s fastest growing large economy, but as we enter 2025, we need to reflect if we are translating our country’s economic wealth into true “wellth” of her people, defined by a life of abundance, purpose and good health.

Although, we have made significant improvements in child health domains such as anthropometric failure, anemia and infectious diseases, the scourge of non-communicable diseases (NCDs) looms large [1]. Based on the data from the Global Burden of Diseases (GBD) study, in 2019, out of 204 countries India has the highest burden of childhood diabetes and childhood diabetes-associated deaths. It is predicted that by 2030, India will have over 27 million obese children, accounting for over 50% of childhood obesity in Southeast Asia, and 10% globally [2]. As of now, 12.5 million children in India are obese already (2022), compared with 0.4 million in 1990 [3].

How are the parents of our kids faring? Out of 828 million people worldwide living with diabetes, an estimated 212 million are Indians [4] Approximately 135 million adults in India have generalized obesity, and 359 million people (40% of the adult population) have abdominal obesity [5]. For comparison, the total population of the US is 340 million!

A recent population-based cross-sectional study among 201 young, healthy adults from Delhi University found that 48.2% of the participants were obese/ overweight, 25% had high waist circumference, 39% had high waist to hip ratio (WHR), 23% had high triglycerides and 23% had hypertension. More than half (50.9%) of the female participants in this study had an elevated WHR [6].

As an Asian Indian immigrant physician in the United States (US) practicing in my endocrinology clinics, I was continually alarmed by the frighteningly early onset of diabesity and its complications in younger and younger individuals. Verily, 90% of all US healthcare dollars, and almost 20% of the US gross domestic product (GDP) is spent on chronic illnesses [7]. To address this catastrophic rise of NCDs, and the spiraling costs of healthcare, the American College of Lifestyle Medicine was formed in 2004. Lifestyle medicine sits compellingly at the intersection of public health and personal wellness and aims to study and implement the evidence behind what constitutes healthy lifestyle.

Almost 90% of type 2 diabetes, 80% of premature heart attacks and strokes, and 40% of cancers can be prevented by adopting simple, low-cost lifestyle measures including healthy nutrition, regular physical activity, restorative sleep, stress management, avoiding use of risky substances, and fostering positive social connections/relationships. The foundation of lifestyle medicine indeed hones in on the etymology of the word “heal” which literally means “to make whole”.

Lifestyle medicine is ***P***roactive, ***P***reventative, ***P***ersonalized and ***P***articipatory (**4Ps**). A practitioner of lifestyle medicine equips and empowers their patients with optimal, scientific tools on how to eat well (addressing the ubiquitous noise and misinformation/ disinformation of influencers/ social media channels), how to improve physical fitness, how to constructively tackle stress through evidence-based techniques like cognitive defusion, Yoga, etc. Unlike acute illnesses, the paradigm of chronic disease is not amenable to management of temporary ills using quick pills, surgical drills and doctor bills. They demand a different skill set, with the practitioner also serving as a lifestyle coach to the patient, much like a sports trainer would mentor a player to achieve their top form and best game. Unsurprisingly, lifestyle medicine has become one of the fastest growing medical specialties in the US [8].

So how can this burgeoning specialty be adapted in the context of the Indian subcontinent? For the prevention of chronic diseases, the Food and Agriculture Organization (FAO) and World Health Organization (WHO), recommend a daily intake of at least 400 g (≥ 5 servings of 80 g each) of fruits and vegetables, wherein the category of tubers (potatoes, cassava) should be excluded. However, a survey done in 2011–2012 showed that the per capita daily household fruit and vegetable intake was 145 and 15 g, respectively, for rural India, and 155 and 29 g for urban India [9], and that majority comprised of potatoes and bananas, with minimal dietary diversity. Lifestyle medicine prescribes a wholesome nourishing plate half-filled with colorful fruits and vegetables (ideally half raw and half cooked), a quarter filled with intact, whole grains (not just wheat and rice, but also millets, buckwheat, whole oat groats, barley, rye, amaranth, etc.) and the remaining quarter filled with diverse legumes, for each meal. Of note, this prescription closely matches a traditional Indian *thali* teeming with flavors, colors, spices and aromas. Unfortunately, with westernization, there is now a trend towards abandoning homemade time-honored culinary offerings for ultra-processed, hyperpalatable calorie dense packaged foods. Similarly, lifestyle medicine practitioners also prescribe replacing harmful, sugar sweetened beverages (SSBs) with plain water or healthy non-caloric beverages like tea. Unfortunately, the sales of aerated drinks and soft drinks in India increased by 22.5% and 24.8%, respectively, from 2016 to 2019 [10], unlike the US where consumption of SSBs has been declining over the last decade [11]. Another harmful trend in Indian dietary patterns is the excessive use of oils and ghee, and unhealthy cooking practices like deep frying and prolonged smoking which destroy valuable nutrients and form deleterious lipid peroxidation byproducts like acrylamides, advanced glycation products, and free radicals. Lifestyle medicine, among its multiple offerings, provides emphasis and training of culinary medicine, to make nutritious yet “unapologetically delicious” (a term coined by Greg Drescher, vice president of the Culinary Institute of America) food. For example, envision a birthday party with oil-free culinary offerings like millet-bean-mushroom burgers on whole wheat buns or air fried sweet potato chips, or beetroot *halwa* cupcakes made without heavy cream or emulsifiers. It cannot be overemphasized that children mirror what their parents eat, and thus, optimal nutrition begins at home.

Exercise has salubrious effects on mental health i.e., improving mood, memory, anxiety, and on physical health i.e., improving blood glucose control, bone mineral density, blood pressure, lowering cholesterol, weight loss, immune function, and fighting cancer. The US Federal Department of Health and Human Services recommend atleast 150 min of moderate intensity aerobic exercises and two or more strength training sessions per week for adults. For children, atleast 60 min of daily aerobic exercises (vigorous intensity atleast thrice weekly) and thrice weekly muscle-strengthening (like climbing or doing push-ups) and bone-strengthening (like jumping or running) activities are recommended [12]. It is also crucial to avoid prolonged sitting and limit screen time. Applying lifestyle medicine, a pediatrician would ideally be able to write a FITT prescription (Frequency, Intensity, Time, Type) e.g. prescribing walk/ bike/ dance/ play cricket (type); daily (frequency); briskly (intensity); for 30 min (time) at 6 PM every evening. For children, the author recommends making it a FFITT prescription (the extra F for friend, e.g. playing cricket with a friend).

Sleep, the third pillar of lifestyle medicine, is perhaps most vital yet often ignored. A study among 657 urban Indian high school-going adolescents, reported that 60% had a sleep duration at night of < 8 h [mean (SD) 7.2 (1.2)] [13]. The increasing use of screens like smartphones and tablets and exposure to blue light in the later parts of the evening causes decline in melatonin levels causing disruption in sleep onset. Sleep deprivation leads to dysregulated metabolism, mood, memory, raging inflammation and over prolonged time leads to developing life-threatening conditions prematurely like diabetes, obesity, infertility, major depressive disorder, cardiovascular disease, high blood pressure, metabolic syndrome and even death. A study in fruit flies reported that accumulation of reactive oxygen species and severe oxidative stress in the gut was the pathway leading to the organism’s death from severe sleep restriction [14]. Similarly, lack of sleep can lead to marked elevation in circulating proinflammatory markers. A randomized controlled clinical trial included 80 young, overweight adults with average age of 29.8 years who were habitually sleeping less than 6.5 h per night at baseline, and randomized them to sleep extension by an average of 1.2 h nightly over 2 weeks. The participants ate almost 300 fewer calories daily due to the increase in their sleep duration and lost on average 0.48 kg in 2 weeks. The researchers projected their findings into the future; eating 270 fewer calories a day would translate to a loss of 12 kg over three years, by simply getting additional sleep [15].

One of the contributing factors to poor sleep among youth is the troublesome alcohol use. A systematic review reported that among Indian medical students (18–23 years) who were drinkers, the prevalence of hazardous drinking was 10–41.5% [16]. In addition to its short-term effects on health like motor accidents, homicides, domestic violence, rape, etc., alcohol use is also associated with long-term effects on human health including cancer. Alcohol is the third leading modifiable risk factor for cancer, particularly, breast cancer, and no dose (including occasional use) can be considered safe. Alcohol had been classified as a Group 1 carcinogen (the highest risk group, which also includes tobacco, radiation, asbestos) by the International Agency for Research on Cancer decades ago, a fact not known to most public leading these authors to comment that alcohol is “cancer’s best kept secret” [17]. Recently, the US Surgeon General Dr Vivek Murthy also issued a health advisory calling for warning labels on alcohol products leading to higher risk of seven types of cancer, akin to tobacco products [18]. Tobacco consumption, in smokeless and smoked forms, remains high in India, particularly the rural and northeastern region. The use of e-cigarettes seems to be rising among Indian adolescents [19]. Worryingly, the prevalence of opioid use in India is three times the global average [20]. About 2.1% of India’s population use opioids. Uttar Pradesh, Punjab and Haryana have the highest number of people with opioid addiction, although in terms of percentage of population affected, the worst affected states are Mizoram and Nagaland. Lifestyle medicine focuses on utilizing evidence-based, multipronged, deaddiction modalities including pharmacotherapy (like varenicline or buprenorphine, disulfiram and perhaps now emerging therapeutic use of glucagon-like peptide 1 receptor agonists etc.), nicotine replacement therapy, cognitive behavioral therapy, dialectical behavior therapy, etc. for these addictions.

Stress is ubiquitous and there never was a time in human history when we did not have to endure stress. However, the coping mechanisms for stress that the human body has designed over millennia of evolution (e.g. activating parasympathetic nervous system to decrease heart rate, blood pressure, improve gut motility etc.) need to be practiced and refined systematically. Practices such as deep breathing, Yoga, meditation, cognitive defusion, etc. all have a significant body of evidence backing their use to improve health conditions ranging from depression to anxiety, to hypertension and diabetes [21]. These techniques and practices will be a vital skill set to survive an increasingly complex world with challenges such as social media, artificial intelligence, climate crisis, threat of drug-resistant microbial pandemics, etc. and our medical schools need to methodically train the future healthcare professional in how to cultivate and conduct a daily routine based upon the foundation of evidence-based mindfulness, awareness and attention training.

Loneliness and social isolation which have been epidemic issues in the first world countries [22], are now worryingly afflicting not only the elderly in India, but also the youth, predominantly due to factors like social media use and substance addiction [23]. Lack of social connections predisposes to 50% higher risk for dementia, 32% higher risk for strokes, 29% higher risk for cardiovascular disease and also significantly higher risk for depression and anxiety. Lack of social connections is the highest risk factor for premature death (50% higher risk) and conversely, healthy relationships can enable people to live longer [24].

To summarize, lifestyle medicine is gaining foothold in the developed economies of the world for addressing the needs of an increasingly unhealthy young population. The Indian Academy of Pediatrics published a timely guideline on the topic of pediatric obesity and recommended many of the pillars of lifestyle medicine as an important foundational measure for preventing and treating this rapidly burgeoning issue [25]. The time is ripe for our pediatric workforce to design, devise, and implement systematic frameworks of prescribing evidence-based lifestyle therapy in clinical practice, health systems and communities.

## Data Availability

Not applicable.

## References

[1] Jain A, Kim R, Swaminathan S, Subramanian SV. Socioeconomic inequality in child health outcomes in India: analyzing trends between 1993 and 2021. Int J Equity Health. 2024;23:149. 39085858 10.1186/s12939-024-02218-z PMC11290299

[2] World Obesity Federation. World Obesity Atlas 2022. Accessed Nov 12, 2024. Available from: https://www.worldobesityday.org/assets/downloads/World_Obesity_Atlas_2022_WEB.pd* f*

[3] NCD Risk Factor Collaboration (NCD-RisC). Worldwide trends in underweight and obesity from 1990 to 2022: A pooled analysis of 3663 population-representative studies with 222 million children, adolescents, and adults. Lancet. 2024;403:1027-50 10.1016/S0140-6736(23)02750-2 PMC7615769 38432237

[4] NCD Risk Factor Collaboration (NCD-RisC). Worldwide trends in diabetes prevalence and treatment from 1990 to 2022: A pooled analysis of 1108 population-representative studies with 141 million participants. Lancet. 2024;404: 2077-93 10.1016/S0140-6736(24)02317-1 PMC7616842 39549716

[5] Anjana RM, Unnikrishnan R, Deepa M, et al. ICMR-INDIAB Collaborative study group. Metabolic non-communicable disease health report of India: the ICMR-INDIAB national cross-sectional study (ICMR-INDIAB-17). Lancet Diabetes Endocrinol. 2023;11:474–89. 37301218 10.1016/S2213-8587(23)00119-5

[6] Mishra S, Murry B, Devi KN, Tripathi S, Suokhrie S. Obesity in dyslipidemia and hypertension: a study among young adults of Delhi/NCR. Clin Epidemiol Global Health. 2023;22:101335.

[7] National health expenditure data: historical. Center for Medicare & Medicaid Services. Updated December 13, 2023. Accessed Feb 6, 2024. Available at: https://www.cms.gov/data-research/statistics-trends-and-reports/national-health- expenditure-data/historical

[8] American Medical Association. Why Lifestyle medicine is growing so fast and the benefits of a lifestyle medicine certification. Accessed Nov 12, 2024. Available from: https://www.ama-assn.org/delivering-care/public-health/why-lifestyle-medicine-growing- so-fast-and-benefits-lifestyle

[9] Minocha S, Thomas T, Kurpad AV. Are ‘fruits and vegetables’ intake really what they seem in India? Eur J Clin Nutr. 2018;72:603–8. 29459786 10.1038/s41430-018-0094-1

[10] Law C, Brown KA, Green R, et al. Changes in take-home aerated soft drink purchases in urban India after the implementation of goods and services tax (GST): An interrupted time series analysis. SSM—Popul Health. 2021;14:100794. 33997244 10.1016/j.ssmph.2021.100794 PMC8102159

[11] Dai J, Soto MJ, Dunn CG, Bleich SN. Trends and patterns in sugar-sweetened beverage consumption among children and adults by race and/or ethnicity, 2003–2018. Public Health Nutr. 2021;24:2405–10. 33843567 10.1017/S1368980021001580 PMC10195631

[12] Centers for Disease Prevention and Control. Child Activity- An overview. Accessed Nov 12, 2024. Available from: https://www.cdc.gov/physical-activity- basics/guidelines/children.html#:~:text=Children%20and%20adolescents%206%20to% 2017,-This%20group%20needs&text=At%20least%203%20days%20a,least%203%20days%20 a%20week.

[13] Mathew G, Varghese AD, Benjamin AI. A comparative study assessing sleep duration and associated factors among adolescents studying in different types of schools in an urban area of Kerala India. Indian J Commun Med. 2019;44:S10–3. 10.4103/ijcm.IJCM_19_19 PMC6824174 31728081

[14] Vaccaro A, Kaplan Dor Y, et al. Sleep loss can cause death through accumulation of reactive oxygen species in the gut. Cell. 2020;181:1307-28.e15. 32502393 10.1016/j.cell.2020.04.049

[15] Tasali E, Wroblewski K, Kahn E, Kilkus J, Schoeller DA. Effect of sleep extension on objectively assessed energy intake among adults with overweight in real-life settings: a randomized clinical trial. JAMA Intern Med. 2022;182:365–74. 35129580 10.1001/jamainternmed.2021.8098 PMC8822469

[16] Nadkarni A, Tu A, Garg A, et al. Alcohol use among adolescents in India: a systematic review. Glob Ment Health (Camb). 2022;9:1–25. 36618747 10.1017/gmh.2021.48 PMC9806994

[17] Slevin T, Chikritzhs T. Why is alcohol cancer’s best-kept secret? Addiction. 2017;112:229–30. 27873368 10.1111/add.13640

[18] U.S. department of Health and Human Service. New surgeon general advisory on link between alcohol and cancer risk. Accessed on Nov 12, 2024. Available from: https://www.hhs.gov/about/news/2025/01/03/us-surgeon-general-issues-new-advisory- link-alcohol-cancer-risk.html

[19] Gupte HA, Chatterjee N, Mandal G, D’Costa M. Adolescents and e-cigarettes in India: a qualitative study of perceptions and practices. Asian Pac J Cancer Prev. 2022;23:2991–7. 36172661 10.31557/APJCP.2022.23.9.2991 PMC9810289

[20] Singh B, Rao R. Is there an opioid epidemic in India? J Public Health. 2021;43:43–50. 10.1093/pubmed/fdab322 34622293

[21] Hempel S, Taylor SL, Marshall NJ, et al. Evidence Map of Mindfulness [Internet]. Washington (DC): Department of Veterans Affairs (US); 2014. Accessed Nov 12, 2024. Available from: https://www.ncbi.nlm.nih.gov/books/NBK268640/25577939

[22] U.S. Department of Health and Human Service. New surgeon general advisory raises alarm about the devastating impact of the epidemic of loneliness and isolation in the United States. Accessed Nov 12, 2024. Available from: https://www.hhs.gov/about/news/2023/05/03/new-surgeon-general-advisory-raises- alarm-about-devastating-impact-epidemic-loneliness-isolation-united-states.html

[23] Pandit TK. A review of loneliness in Indian youth. Int J Indian Psychol. 2021;8:406–13.

[24] Harvard Medical School. Study of adult development. Accessed on Nov 12, 2024. Available from: https://www.adultdevelopmentstudy.org/grantandglueckstudy

[25] Khadilkar V, Shah N, Harish R, et al. Indian academy of pediatrics revised guidelines on evaluation, prevention and management of childhood obesity. Indian Pediatr. 2023;60(12):1013–31. 38087786

