# Erratum: Lifestyle Medicine and the Role of the Pediatrician

**DOI:** 10.1007/s13312-025-00125-2

**Published:** 2025-06-20

**Authors:** Mahima Gulati, Suneela Garg

**Affiliations:** 1 Department of Medicine, Division of Endocrinology, Diabetes, and Metabolism, University of Connecticut Health School of Medicine, Farmington, CT USA; 2 Community Medicine, MAMC, Delhi Chair, Programme Advisory Committee, NIHFW, Delhi, India

**Correction to: Indian Pediatrics** 10.1007/s13312-025-00066-w

In this article, affiliation 2 “Community Medicine, MAMC, Delhi Chair, Programme Advisory Committee, NIHFW, Delhi, India” was incorrectly written as “HIV Medicine Regional Training Centre, Maulana Azad Medical College, New Delhi, India.”

 The original article has been corrected.

